# Temperature and voltage effects on the charge and health of lithium-ion battery modules in light electric vehicles

**DOI:** 10.1038/s41598-026-40094-5

**Published:** 2026-02-17

**Authors:** Jessica Meza Quintana, Juan Carlos Paredes-Rojas, Rubén Vázquez-Medina, Omar Jiménez-Ramírez, Christopher Rene Torres-San Miguel

**Affiliations:** 1 Instituto Politécnico Nacional, Escuela Superior de Ingeniería Mecánica y Eléctrica Unidad Culhuacán, Santa Ana 1000, Coyoacán, CTM Culhuacán, 04440 Mexico City, Mexico; 2 Instituto Politécnico Nacional, Centro de Investigación en Ciencia Aplicada y Tecnología Avanzada, Unidad Querétaro, Cerro Blanco 141, Col Colinas del Cimatario, 76090 Querétaro, México; 3 Instituto Politécnico Nacional, Escuela Superior de Ingeniería Mecánica y Eléctrica, Unidad Profesional “Adolfo López Mateos” Gustavo A. Madero, Col. Lindavista, 07738 Mexico City, Mexico

**Keywords:** Lithium-ion battery, Light electric vehicle, State of charge, State of health, Thermographic test, Energy science and technology, Engineering, Environmental sciences, Materials science

## Abstract

Lithium-ion batteries are an alternative to mitigate environmental impacts in the vehicle sector. However, their performance is limited by degradation during use, downtime, and environmental and operating conditions. This work analyzes the behavior of lithium-ion batteries when used in a light electric vehicle. Specifically, it determines the state of charge and state of health of a lithium-ion battery package when used in a light electric vehicle with a 500–1080 W power rating and a 48 V, 8.8 Ah electric motor. This light electric vehicle underwent a worldwide harmonized motorcycle emissions certification/test procedure, while the lithium-ion batteries were evaluated at 25, 35, 45, and 65 °C. Voltage, amperage, and temperature were measured in real time. A thermographic study was also conducted in which heat transfer images of lithium-ion batteries were taken in different positions. The results show that the lithium-ion battery package retains more heat in the central area of the module. For example, in the 25 °C test, the most critical temperature reading was 29.9 °C. In the 65 °C test; however, the most critical temperature reading was 80.2 °C. The implementation of a ventilation system for the lithium-ion battery package for applications in light vehicles was evident. Lithium-ion batteries were found to be sensitive to temperatures above 35 °C. These high temperatures cause accelerated voltage loss and an imbalance in cell charge, which consequently lowering performance. Therefore, the state of charge and state of health of the Lithium-ion battery package are significantly impacted.

**Supplementary Information:**

The online version contains supplementary material available at 10.1038/s41598-026-40094-5.

## Introduction

The battery is a device that produces and stores electrical energy through chemical reactions^1^. The advancement of lithium-ion batteries (LIBs) began during the late 1950s when military and space operations required smaller batteries, but with an improvement in battery density compared to existing ones^2^. In 1991, SONY Corporation launched the first commercial LIBs^3^. Since then, LIBs have become outstanding and widely used energy storage technology in electronic products^4^.

**Fig. 1:**
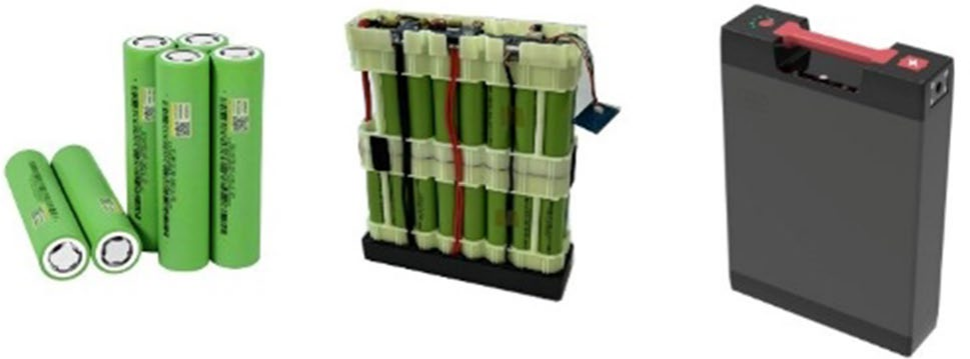
Cell, module, and battery pack^8^.

Currently, LIBs serves as an energy storage solution that helps electrify transportation^5^. One of the main objectives is to reduce the pollutant gasses emitted by vehicles. However, battery life remains limited due to degradation that occurs during use and inactivity. The battery’s lifespan is also affected by its structure, operation, and the environmental conditions in which it is located^6,7^. As shown in Fig. 1, the battery is grouped into three different levels.

The correct estimation of battery lifespan is crucial for the development of device technologies. Platforms are feasible tools for simulating battery models. For example, Brondani et al.^9^ proposed an algorithm for parameterizing a battery model using the MATLAB platform. They parameterized and validated the model using experimental data obtained from a test bench.

The maintenance of the health of LIBs is of great interest, leading to the development of various research topics that extend their useful life. For instance, thermal management is an essential issue for ensuring thermal stability and long-term life of the LIBs. For this reason, in the research Yu et al., proposed a thermal management system. That system consisted of two types of air duct with independent inlet channels and fans. Then, a heat transfer model is developed to describe the thermal behavior of the LIBs^10^.

An important process in any numerical analysis is the meshing process, which involves subdividing the geometry into numerous small control volumes, or elements. As Yousefi, et al., demonstrates in his study where he used 3 different types of meshes containing: hexahedral, tetrahedral, and polyhedral elements. To model the heat generation within a LIB, he adapted an electrochemical-thermal model. The maximum temperature of the battery exceeded the optimal range of the cell 40 Celsius degrees (°C), demonstrating that more efficient passive techniques are required to cool the cell^11^.

Accurately determining the state of charge (SOC) is becoming increasingly important because it optimizes battery performance. Several factors must be considered, such as achieving full charge to maintain battery life and determining the point closest to full discharge to ensure controlled device shutdown and prevent potential data loss or damage to the electronic system. Having an accurate method for assessing the SOC of batteries is equally important^12^.

Charging capacity has thus become one of the key characteristics of the battery and electric vehicle industries. However, according to Chen et al.^13^, in their study, rapid charging accelerates battery degradation. As LIBs age, their performance tends to decline. Therefore, current methods still require laboratory tests to determine the rate and extent of this decline. A method developed by the University of Warwick allows for rapid and accurate assessment and ensures the validation of the state of health (SOH) and SOC of the battery. The true determining factor is the battery age^14^. Several authors have mentioned that LIB’s can present a risk of smoke, fire, or electric shock if mishandled. Therefore, a battery management system (BMS) monitors most of the analyzed battery variables (temperature, overload, state of charge, and others)^15^.

Thermal analysis is a highly relevant topic regarding batteries. Understanding how temperature affects batteries is key to maintaining good battery health. However, battery chemistry behaves differently at high or low temperatures. Batteries perform best at room temperature (25 °C). However, their temperature may increase or decrease depending on where they are installed. Batteries tend to perform better at higher temperatures due to reduced internal resistance and accelerated chemical reactions. However, these conditions accelerate the corrosion caused by the electrolyte, shortening their useful life^16^. Evaluating a battery SOH allows to determine its suitability for reuse (second life). In simple terms, the state of health (SOH) of a battery is the difference between the initial state of a new battery and its state after use. This is represented as the percentage of energy capacity a battery can store, which decreases over time^17^. The SOC represents the relationship between the amount of charge that can be extracted from the cell within a specific time frame and the total capacity. The SOC estimation of the battery informs users of the usable capacity until the next recharge, keeps the battery within the safe operating threshold range, implements control strategies, and helps extend the battery life^18^. Recently in the College of Mechanical and Vehicle Engineering, Chongqing University, a battery pack 3 Parallel, 4 Series (3p4s) was used considering an electrical model that provides electrical parameters for a thermal model that established a detailed framework for heat generation and heat transfer in the battery pack. This approach allowed to correct the values of some parameters. The SOC of each unit in the pack was estimated and edited-corrected using state of temperature (SOT). A heat generation model was used to calculate the SOC, which then allowed to determine the central temperature of each cell. In addition, an accurate 3D temperature distribution was obtained; thus, the proposed method demonstrated good accuracy and reliability under various operating conditions and temperatures, and imaging thermal revealed the highest heat concentration in the central area^19^.

Another relevant method was demonstrated in the study by Hosseion, et al., in that study, a numerical model was developed to describe the interaction between different finned configurations along with local oscillation within a rectangular finned container^20^.

Batteries are sensitive to high and low temperatures, which causes accelerated degradation. In this regard, Xinqi, et al., conducted a study proposing two degradation mechanisms of battery that quantitatively decouple from the normal aging process. In this case, it was demonstrated that plated lithium can be generated. In addition, the study proposed the use of combined protocols was proposed to significantly accelerate the battery degradation process. From an experimental point of view, normal aging in 80% SOH required 1400 cycles, theoretically consuming 4900 h (hrs.)^21^.

On the other hand, Seyed et al.^22^ found that exposing batteries to high temperatures can cause uncontrolled overheating and accelerated degradation, which negatively impacts performance and safety. The main strategies to minimize this degradation include optimized charging protocols, controlled depth of discharge, and the implementation of a thermal management system.

LIBs are used in light electric vehicles (LEV) such as bicycles, motorcycles, and electric scooters. This allows the operator to achieve greater autonomy with less physical exertion. Electric vehicles (EV) are classified as low, medium, or high power measured in watts (see Table 1).

**Table 1:** Classification of EV^23^.

Low consumption	Medium power	High power
Less than 250 W	Between 250 and 500 W	More than 500 W
Light and their use is in the city	It is used for a city and long journeys	They travel through difficult terrain or extreme routes

The standard power for EV is 250 Watts (W), which is the limitation imposed by the European Union to avoid the need for approval, registration, or insurance to operate them on public roads^24^. Based on previous studies, incorporating LIBs into EV poses several challenges, including maintaining an adequate operating temperature for batteries, preventing premature aging, and avoiding imbalances to ensure proper operation.

The state of the art in recent scientific studies reveals that thermal analysis is of great importance in the field of batteries. Understanding how temperature affects batteries is key to maintaining their optimal condition. However, their temperature can increase or decrease depending on their location and external factors. Batteries tend to function better at higher temperatures due to lower internal resistance and the acceleration of chemical reactions, but this also shortens their lifespan. Furthermore, charge capacity has become a key characteristic in the battery and electric vehicle industries. Evaluating the State of Health (SOH) of a battery is crucial for determining its suitability for reuse.

This work proposes and analyzes the use of LIBs. SOC and SOH behavior of a LIB were experimentally determined. The tests were carried out on a LEV with a power output of 500–1080 W, a 48 Volts (V) electric motor, and a LIB of 48 V and 7 Ampere hour (Ah). Its maximum speed was 56 Kilometer per hour (km/h) and its maximum load was 150 kg (kg). The LIB was subjected to temperatures of 25 °C, 35 °C, 45 °C and 65 °C, using a World-wide Harmonized Motorcycle Emissions Certification/Test ProCedure (WMTC) certified driving cycle. Voltage, amperage, and temperature were taken after each test to determine the behavior of SOC and SOH in each cell. A thermographic study was also conducted, and heat transfer images were taken at different positions of the LIBs. Based on these studies, a natural convection cooling system was designed.

Therefore, this work is structured as follows. Section “Batteries” describes the characteristics, classification, and types of LIBs, as well as its SOC and SOH. Section “Methodology” describes the methodology, materials, and equipment used. Section “Results” presents the results and discussion of experimental tests on the SOC, SOH, and temperatures of the battery. Finally, Section “Discussion” presents the conclusions and possible future work.

## Batteries

Proper battery operation requires an accurate assessment of the SOH and SOC of the battery. An effective BMS is also necessary for optimal management of the battery. SOC and SOH are two key metrics used in BMS to provide data on the existing charge and overall condition of the battery^25^. However, accurately monitoring battery SOC and SOH status is complicated by the way in which the battery’s internal chemical reactions and physical processes respond to various nonlinear voltages and currents^26^. The capacity of a battery is commonly C-rate at 1C, which means that a fully charged battery rated at 1Ah should provide 1Ampere (A) for one hour^27^. The design of the cooling system, the weight, and its size are directly influenced by the geometry of each cell^28^.

It is defined as the ratio of the measured capacity to the nominal capacity. The ideal state of health is 1, when both capacities are equal and the battery is new. By definition, a battery is at the end of its useful life in a SOH of 0.8^29^.

SOH refers to the ratio between the characterization parameters of LIBS’s (e.g., battery capacity) to that of which when they are not used, which is given in percentage^30^. This percentage represents the initial capacity^31^ and reflects the current available capacity of the battery. Generally, it is defined as the ratio of the current maximum capacity to the nominal capacity^32^, which can be calculated using Eq. (1).

1 $$SOH = \frac{Qcu}{{Q_{e} }} \times 100\%$$

where $Qcu$ is the actual maximum capacity [Ah] and $Qe$. rated capacity [Ah].

accurate assessment of the battery SOC allows for an accurate evaluation of the reing running time of the car and for managing the battery^26,33^. For example, when a battery is completely charged and when it is completely discharged. See Eq. (2).

2 $$SOC\left( \% \right) = \left( {\frac{Qre}{{Q_{cu} }}} \right) \times 100$$

where $Qre{ }$. remaining capacity [Ah] and $Q_{cu}$ current maximum capacity [Ah].

## Methodology

The experimental tests were conducted according to the IEC 61982 Standard^34^, where tests and requirements are specified. These tests can be carried out on batteries for applications in LEV, motorcycles, commercial vehicles, etc.^32^.

**Fig. 2:**
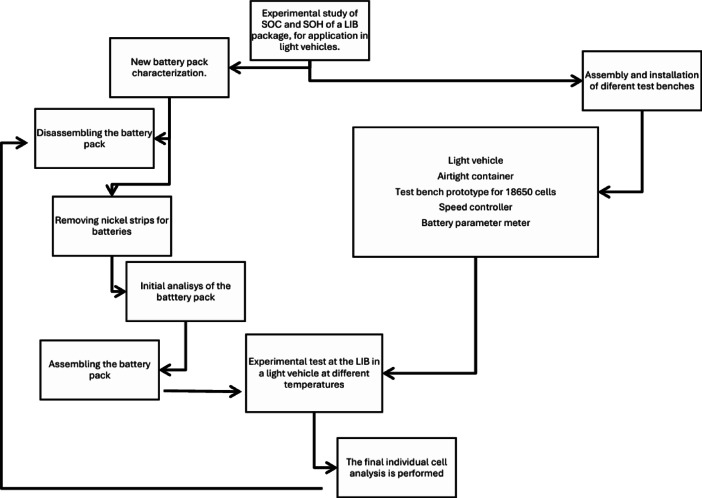
Methodology of this experimental tests of SOC and SOH in the LIB in a LEV.

**Fig. 3:**
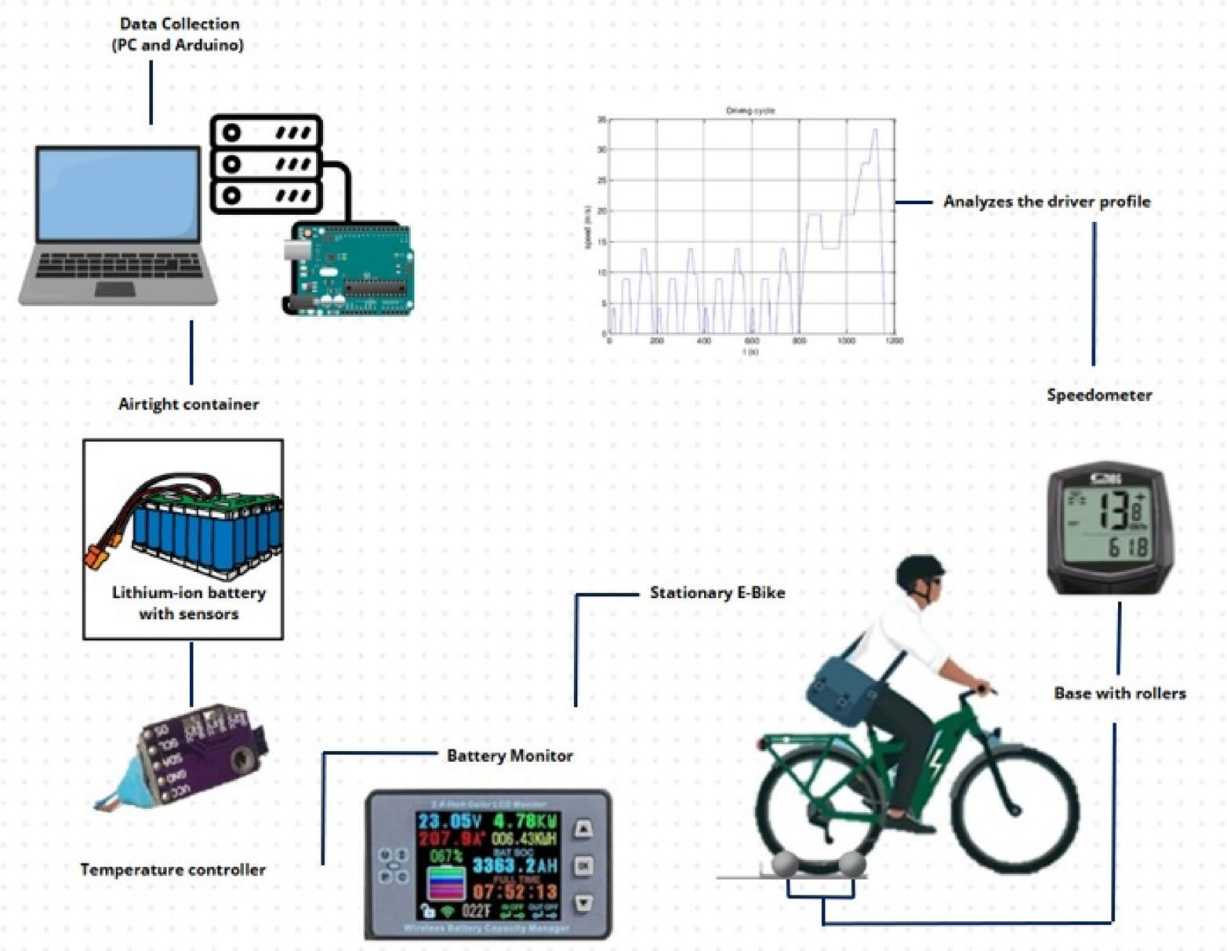
Diagram of the LIB experiment.

The methodology of this experimental study was conducted in different stages, as shown in Figs. 2 and 3.

Figure 2 shows the stages of the experiment. As a first step, the characteristics of the battery module were determined and investigated (voltage, current, temperature range, and cell connections). Subsequently, it was disassembled, and the nickel strips connecting each cell were removed. The cells were then separated individually and analyzed on the test bench, thereby obtaining the initial values (SOC and SOH), considering that they were new cells. Once the individual cell analysis was completed, the battery module was reassembled.

For the experiment, a hermetically sealed thermal container was designed and assembled, along with a test bench for 18,650 cells that allows for the individual charging and discharging of each cell. A reader was also designed to obtain real-time battery parameters (voltage, current, temperatures), as well as data from the LEV installation and the battery module connection. A speed controller was also designed and assembled, programmed with the WMTC conduction profile. These devices are detailed later.

The experiments were conducted at different temperatures (25, 35, 45, and 65 °C). Upon completion, the cells were disassembled and individually analyzed again. After all the tests were finished, the module was disassembled and the cells were analyzed once more on the test bench.

Figure 3 shows the equipment, instruments, and tools used during the experiment. It started with the LEV located on the rollers, which allowed the tires to rotate at different speeds. This LEV works with a LIB, which is housed in an airtight container where the ambient temperature is controlled with the help of an electric heater. Parameters such as voltage, current, and temperature were monitored with a real-time battery monitor. Finally, temperature sensors were installed at specific points in the LIB. An ESP32 microcontroller was used to program the WMTC driving cycle. All the information obtained was automatically recorded on a computer.

### Materials and equipment

In this experimental test, a lightweight vehicle with a rated power of 500 W–1080 W (see Table 2) was used. This vehicle was mounted on a roller platform that allowed the wheels to move freely, achieving low and high speeds. This vehicle was subjected to an experimental driving cycle. Nomenclature can be found in the supplementary material.

**Table 2:** LEV specification.

Technical details	
Material	Aluminum alloy
Unfolded dimensions	170 × 64 × 120 Centimeters (cm)
Equipment weight	38 kg (kg)
Maximum load	150 kg
Maximum speed	56 Kilometer per hour (km/h)
Range	45 km (km)
Rim size	20 inches
Motor	
Maximum power	1080 W
Nominal power	500 W
Nominal voltage	48 V
Riding modes	
1. Pedal-only riding mode	
2. Assist mode	
3. Purely electric mode	

**Fig. 4:**
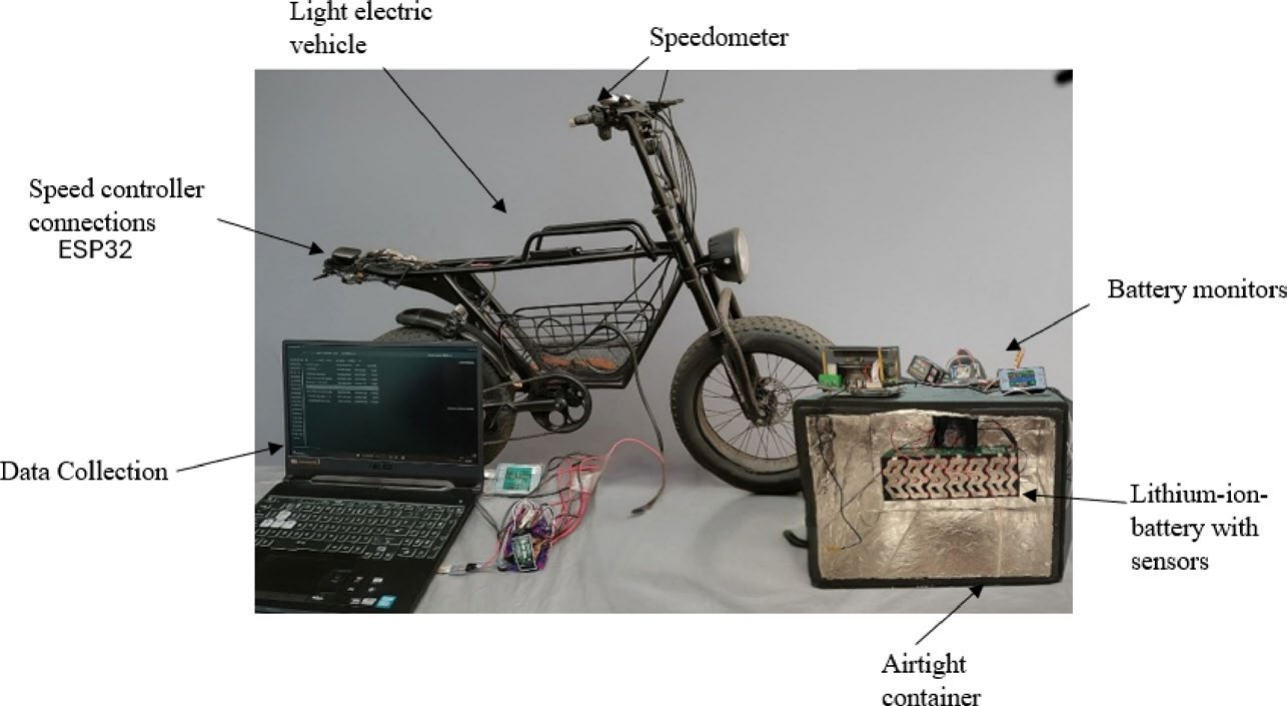
Equipment, instruments, and tools.

Figure 4 shows a hermetic container, which is a prototype for experimental temperature tests of the battery module with a temperature variation of 25 to 65 °C. On the left side of Fig. 4, the state of charge of the batteries is measured by using a computer and a data acquisition system. A temperature controller is used to regulate the heater to maintain the required temperature. This controller has a K-type thermocouple with a Max6675 module that records the temperature inside the container using an ESP32 programming board. K-Type thermocouples are characterized by their wide temperature range, with capabilities as low as − 200 to + 1250 °C. Made of chrome and aluminum alloys, they are distinguished by a positive yellow and negative red color code.

A prototype was designed to charge and discharge 18,650 LIB cells. The prototype used a ZB2L3 discharge tester and a TP4056 charging module. The TP4056 is a complete constant-current/constant-voltage (CC-CV) linear charger for single cell lithium-ion batteries. Furthermore, the TP4056 can work with USB and wall adapters. Thermal feedback regulates the current charge to limit the die temperature during high-power operation or high ambient temperature. The charge voltage is fixed at 4.2 V, and the charge current can be programmed externally with a single resistor. After the start of the test, the tester ZB2L3 will control the load of the electronic switch that is turned on. The test data shows that the process will release capacity (Ah), current discharge current (A), and battery voltage (V) between the wheel. When the battery voltage reaches the set cut-off voltage, the load control switches off, and the tester display data retains the capacity (Ah) and above, and the corresponding indicator flashes quickly, displaying that the battery is discharging. The prototype has 20 discharge testers and 24 charging modules. These experimental tests were performed at the beginning and end of the temperature tests; that is, the cells were subjected to different working temperatures. The module was subsequently disassembled to free each cell for measurement. The modules and testers are connected to a 5 V, 30 A switching power supply.

**Fig. 5:**
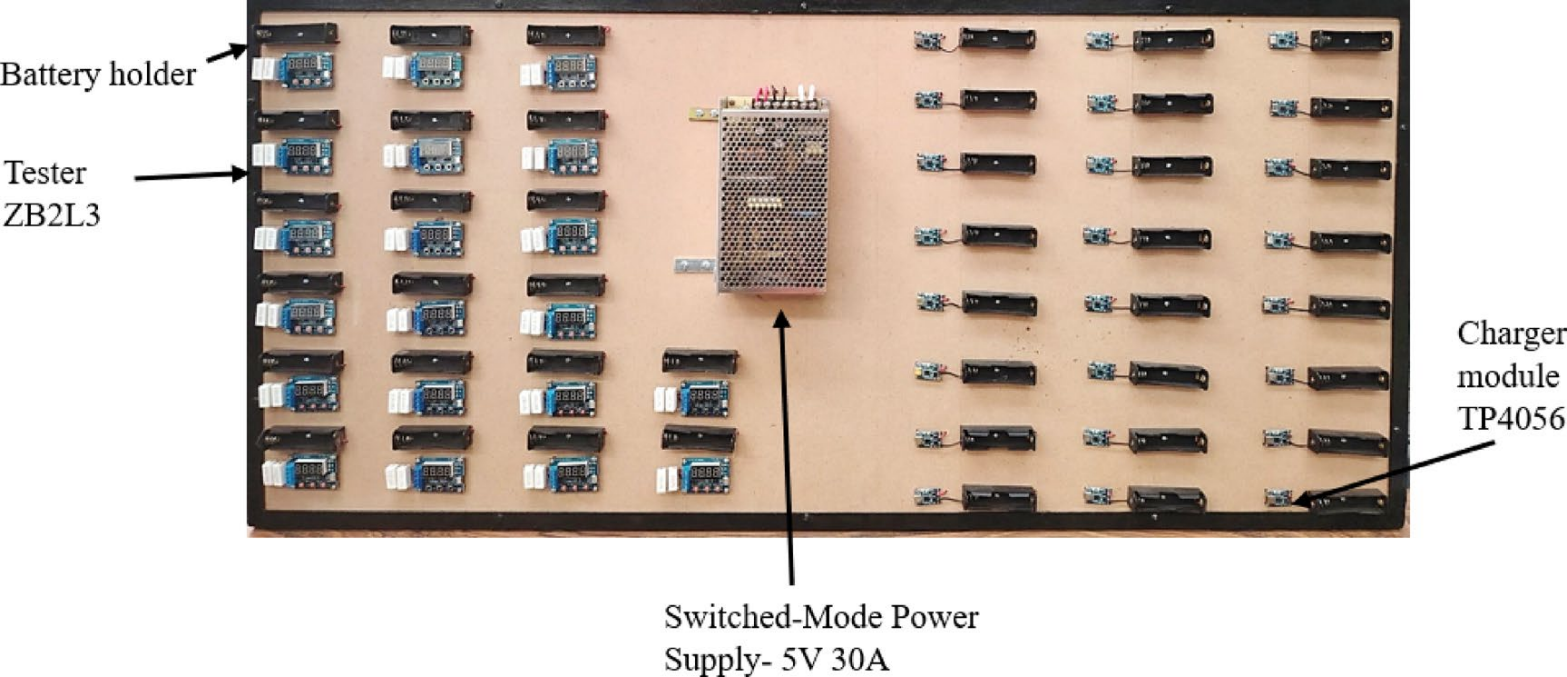
Discharge and charge modules for 18,650 lithium-ion batteries.

The following Fig. 5 shows the design of the prototype for loading and unloading tests.

The WMTC driving cycle was programmed using an ESP32 microcontroller.

**Fig. 6:**
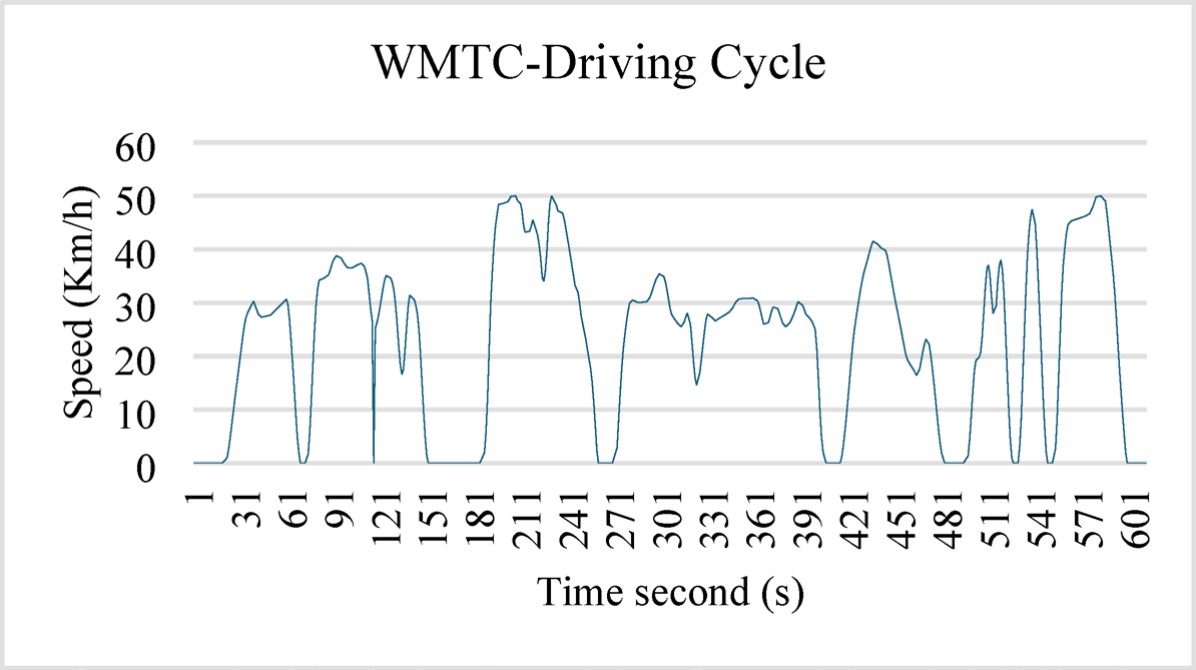
Driving cycle.

Figure 6 shows the WMTC driving cycle; this is a standardized cycle with which tests are performed on LEV^35^.

**Fig. 7:**
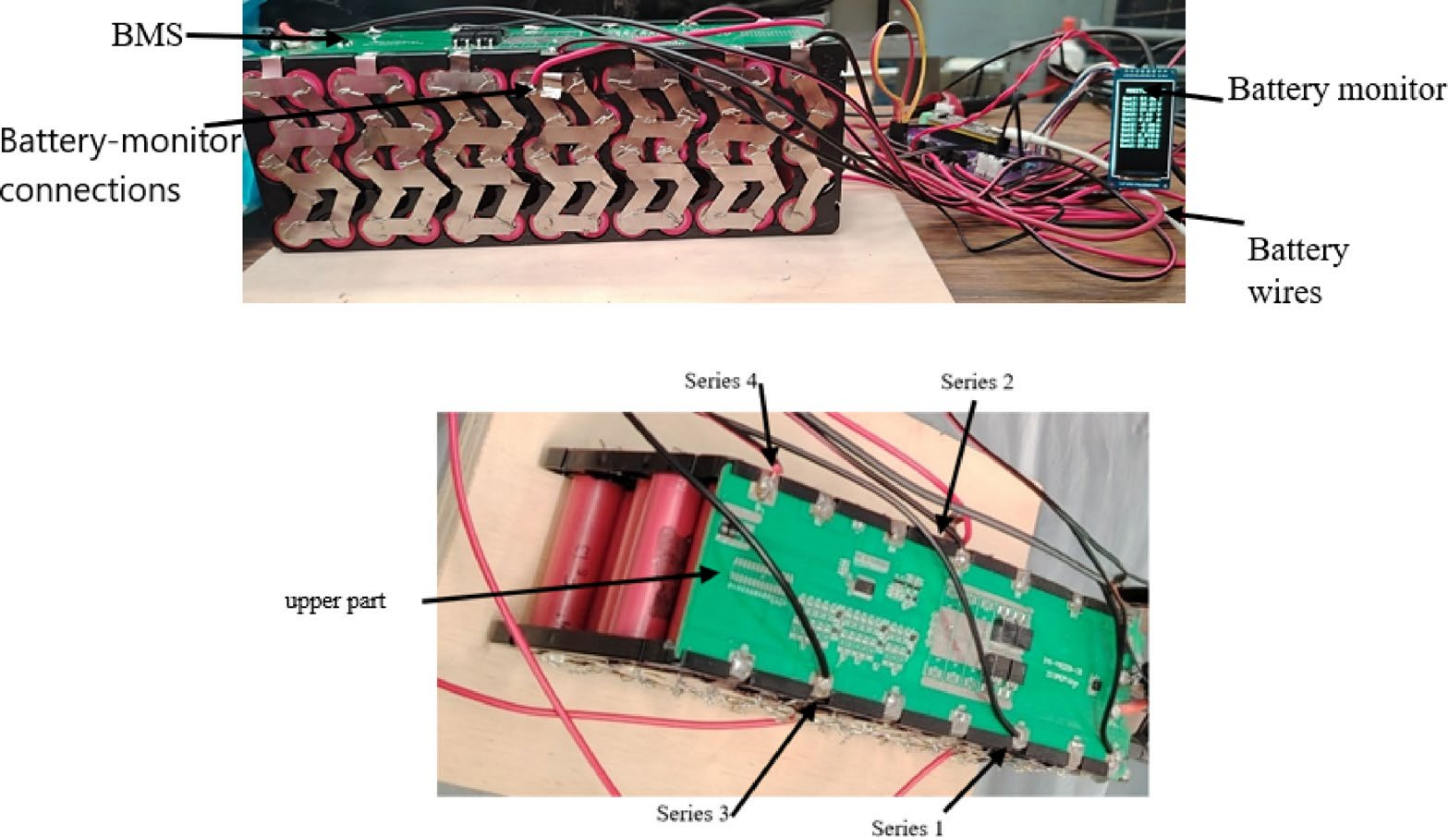
Battery measurement reader.

Figure 7 shows the installation of a battery parameter measurement module; this allowed monitoring the battery behavior in real time during its use in the LEV. This module measures characteristics such as voltage, temperature and amperage. The LIB module was analyzed in four series: series one has 3 cells, series two has 6 cells, series three has 9 cells, and series four has 13 cells (12.4, 25.6, 37.2 and 54.6 Volts, respectively).

The battery module features a nickel foil structure that connects the cell electrodes to the BMS. The temperature in this structure was not considered because the electrode thickness is small and does not significantly affect heat transfer.

**Fig. 8:**
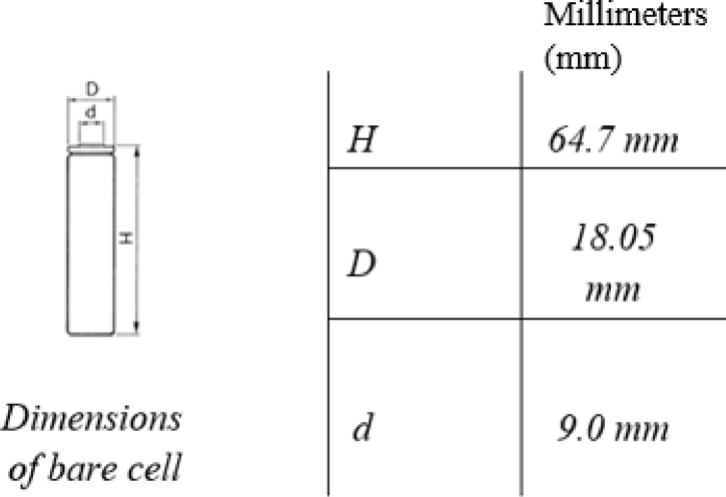
Characteristics of LIB 18,650 cells.

The LEV was powered by a battery module with the following characteristics (see Tables 3, 4, and Fig. 8).

**Table 3:** Battery pack characteristics.

Nominal voltage	48 V
Ability	8.8 Ah
Type of arrangement	13 s 4p
Cell type	18,650

**Table 4:** Cell type UR18650A.

Specifications	
Manufacturer	SANYO
Nominal voltage	3.6 V
Nominal capacity (mAh)	Min. 2 150 Milliamp hour (mAh)
Charging method	Constant Currente-Constant Voltage (CC-CV)
Charging voltage	4.2 V
Charging current	Std. 1 505 Milliamp (mA)
Charging time	3.0 h
Anode	Graphite
Cathode	Lithium oxide
Weight (Max.)	43.0 g (g)
Chemistry	Lithium ion
Ambient temperature	
Charge	0 ~ + 40 °C
Discharge	− 20 ~ + 60 °C
Storage	− 20 ~ + 50 °C

**Fig. 9:**
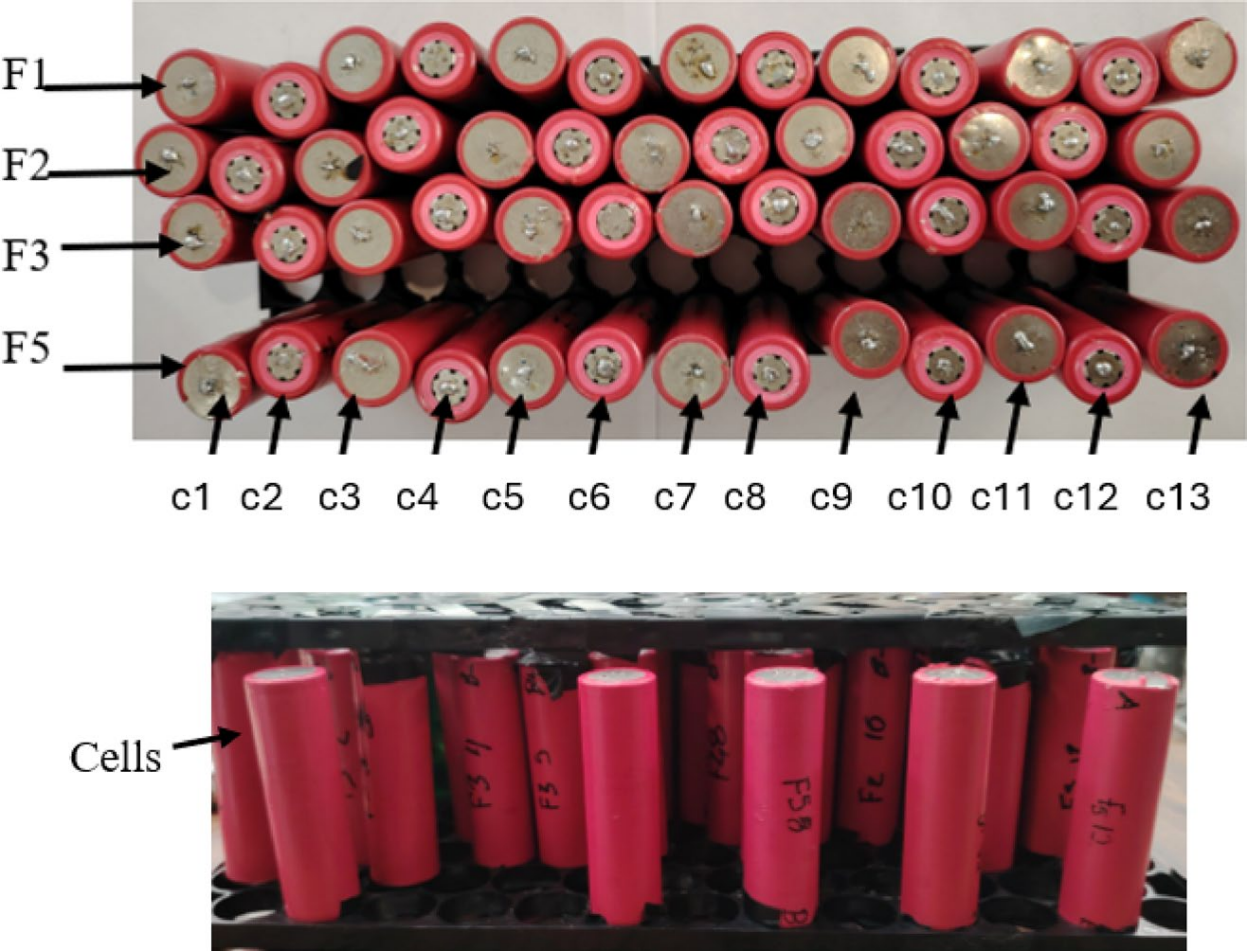
18,560 cells that make up the battery module.

The battery module has 52-18650 cells, as shown in Fig. 9, which are joined with nickel tape using spot welding.

Cells are numbered in rows and columns (see Fig. 9), where: F1: row 1, F2: row 2, F3: row 3, F5: row 5, c1: column 1, c2: column 2, c3: column 3, c4: column 4, c5: column 5, c6: column 6, c7: column 7, c8: column 8, c9: column 9, c10: column 10, c11: column 11, c12: column 12, and c13: column 13.

Considering that the central cells are F2 and F3, the relevant cells are 5, 6, 7, and8.

The prototype test bench (Fig. 5) was designed and built to analyze each cell, specifically the charge and discharge of each cell, to obtain the actual capacity in ampere hours. As a first step, the battery module (new, unused equipment) was analyzed at room temperature (23 °C). The SOC and SOH of each cell were determined; this data served as a reference point for the experimental tests.

The battery module is connected to a charger with an output of 54.6 V–2.0 A.

The LIB was subjected to different temperatures in the hermetic container: 25, 35, 45, and 65 °C, respectively. It is important to mention that each temperature test lasted 30 min, and the battery was allowed to rest to reach its resting state. The LIBs were charged according to the test temperature; subsequently, measurements were obtained from the LIB module every five seconds in real time during the driving cycle. Finally, the module was disassembled for cell-by-cell SOC and SOH analysis. The results for each cell were compared between the initial and final states to determine their actual state.

The following equipment errors were demonstrated (Table 5).

**Table 5:** Error ranges.

Equipment	Error range
Tester ZB2L3	1% + 0.02 V 1.5% ± 0.008A
Controller REX-C100	± 2 °C
Thermocouple type K with a Max6675 module	± 2 °C

## Results

**Fig. 10:**
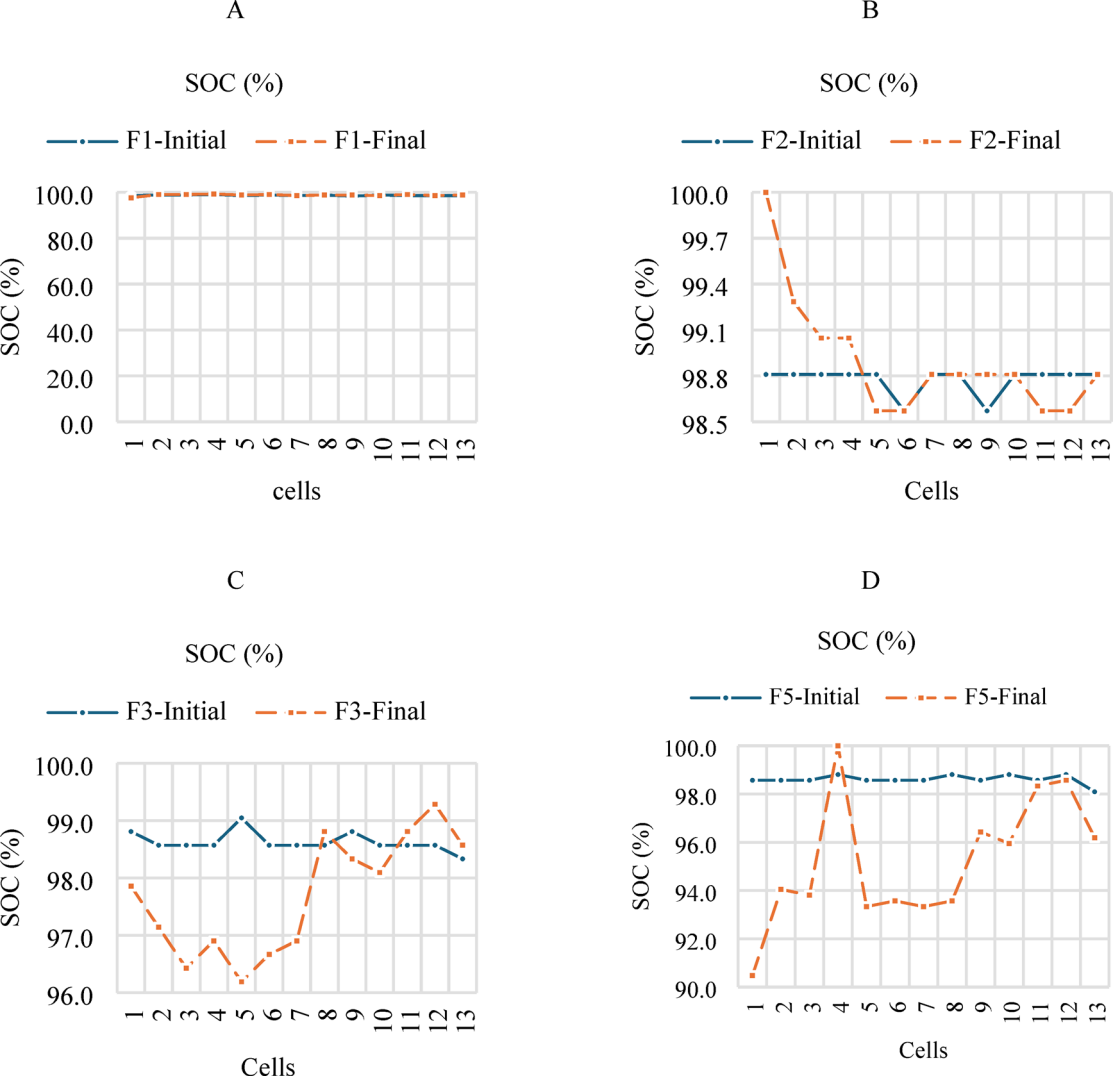
SOC results of LIB’s. A. F1, B. F2, C. F3 and D. F5.

Figure 10 shows four graphs, A, B, C, and D, showing the actual capacity of each cell in the LIB module. Fx-Initial refers to the values obtained in the first analysis with the new cells, and Fx-Final refers to the values ​​obtained after all the tests performed. Cells were found to have low values (initial F1, F2, F3, and F5) relative to their original capacity. This may be due to various factors, such as transportation, storage, and/or manufacturing date. After testing, each group F1, F2, F3, and F5 shows that some cells lost significant capacity, which affects performance. These cells are the farthest from the BMS and the central part of the module. The central cells are the most affected in their SOC value.

**Fig. 11:**
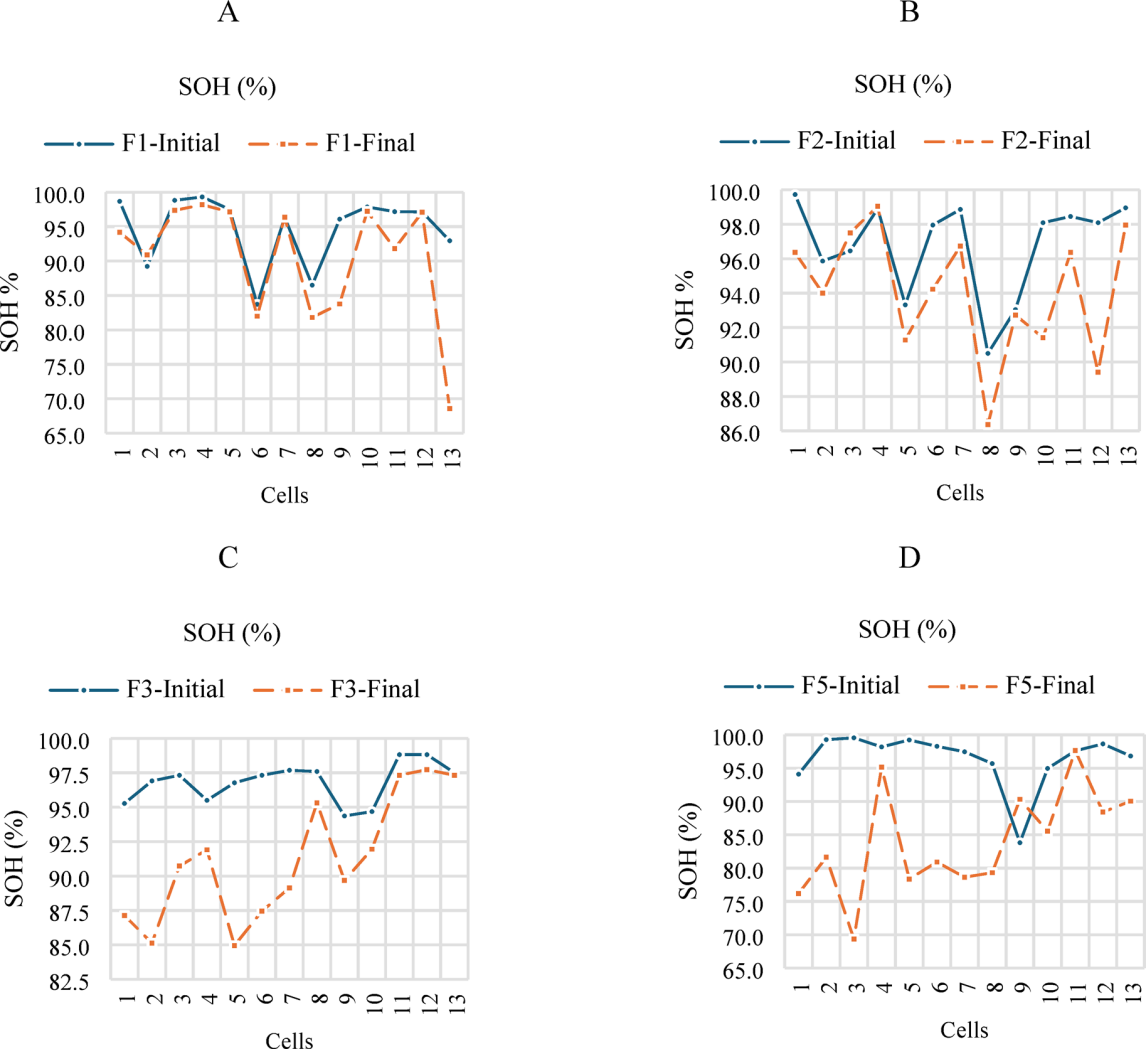
SOH results of the LIB. A. F1, B. F2, C. F3 and D. F5 initial and final.

Figure 11 shows the battery SOH results; it is observed that F3 and F5 present significant degradation. To be precise, some final battery SOH values are below 80%, with respect to the Institute of Electrical and Electronics Engineers (IEEE) 1188–1996 standard, and this suggests a battery change^35^. However, this time the level is mainly affected by the charge temperature above 35 °C, particularly in the 65 °C charge, where an imbalance was caused. A SOH level between 70 and 80% can be considered the first phase of the battery’s lifespan for certain applications. In some cases, batteries can be reused for a second life in smaller electronic devices that require lower power consumption^36,37^. In this study, batteries degrade because of exposure to high temperatures, which causes an increase in the chemical activity within the battery and accelerates its degradation. However, there are other factors that can also cause them to age.

**Fig. 12:**
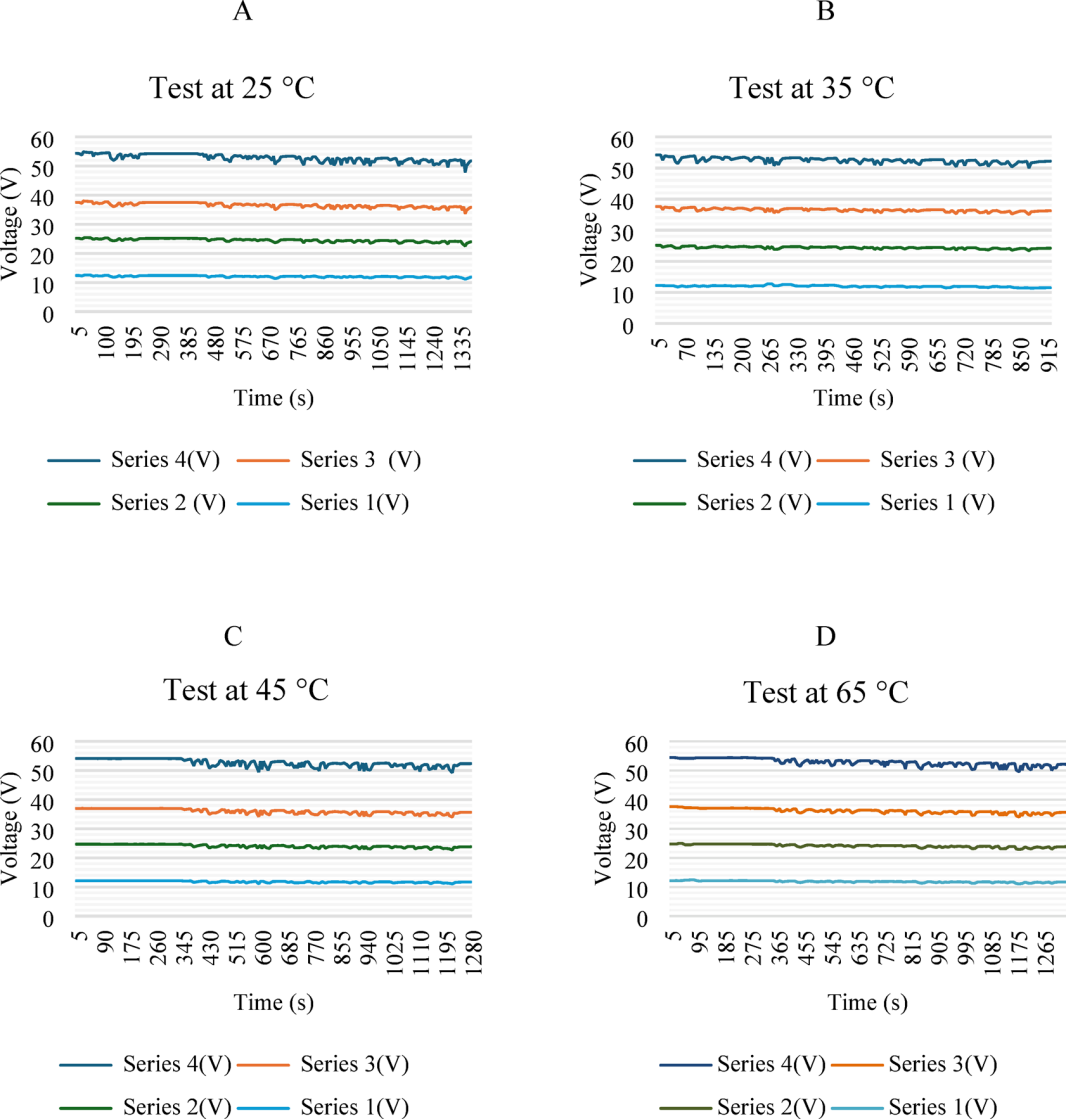
Voltage behavior in conduction cycle.

Figure 12 shows four graphs depicting voltage behavior as a function of the driving cycle. The module was analyzed in four series: 12.6, 25.6, 37.8, and 54.6 V, respectively. Series 1 and 4 represent the lateral parts of the module, while series 2 and 3 represent the central parts. A slight decrease in voltage is shown because the vehicle is in a driving cycle. Similar voltage consumption is shown at temperatures of 25, 35, and 45 °C, with a slight decrease in voltage as the temperature increases. However, at 65 °C, greater voltage loss was observed.

**Fig. 13:**
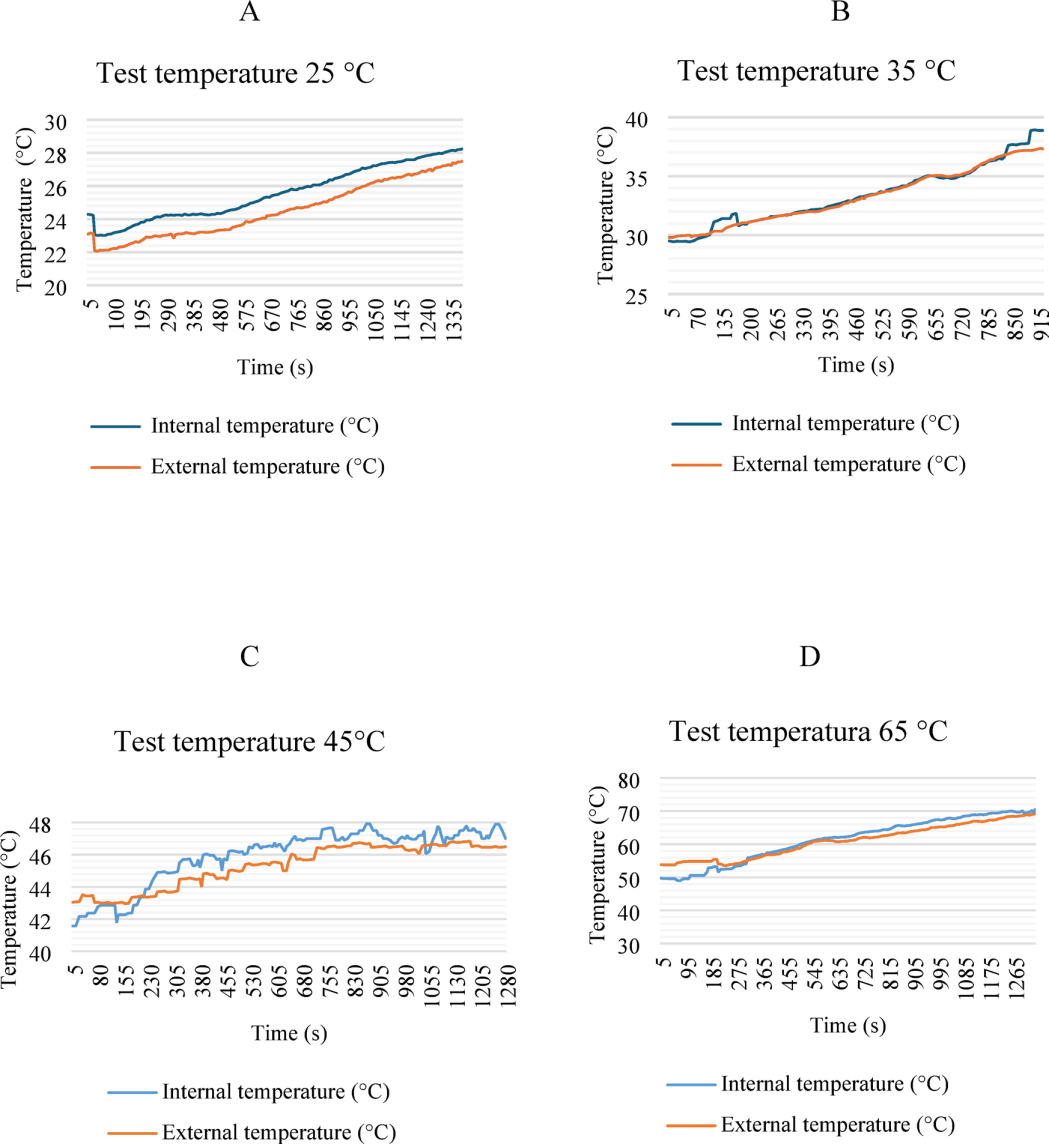
LIB temperatures in operation with the driving cycle.

Figure 13 shows the temperature results of the LIBs as a function of the driving cycle; two temperature sensors were placed, one on the side and one located inside the module. In the 25 and 35 °C tests, there was a slight increase in temperature since the driving cycle occasionally requires maximum speeds. Even so, the battery is within suitable temperature ranges for proper operation. However, at temperatures of 45 °C and 65 °C, the temperature increases considerably, which directly affects the battery’s lifespan and can cause thermal runaways.

**Fig. 14:**
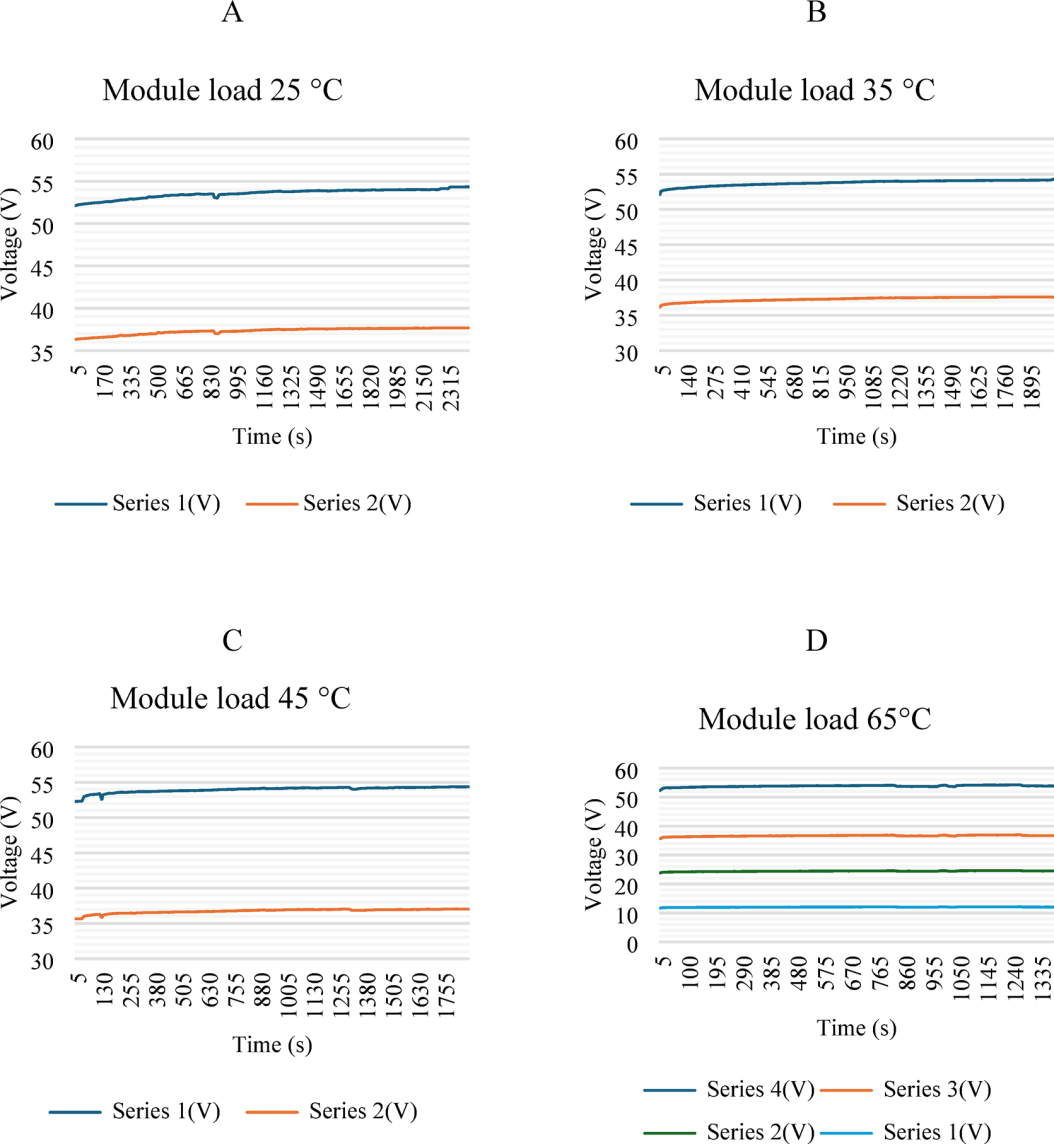
Voltage behavior during the charging process.

Figure 14 shows the charging behavior of the LIB’s. In this test, the LIB module was disconnected from the LEV, which means that the module was temperature controlled during charging. At 25 °C, the module was properly charged, reaching 54.2 V. At 35 and 45 °C, slight variations occurred due to the increased temperature. Nevertheless, the module still managed to charge properly, reaching 97 to 99% (SOC). Conversely, the battery failed to fully charge at 65 °C. In these cases, the BMS protects the battery from high temperatures but also causes an imbalance.

**Fig. 15:**
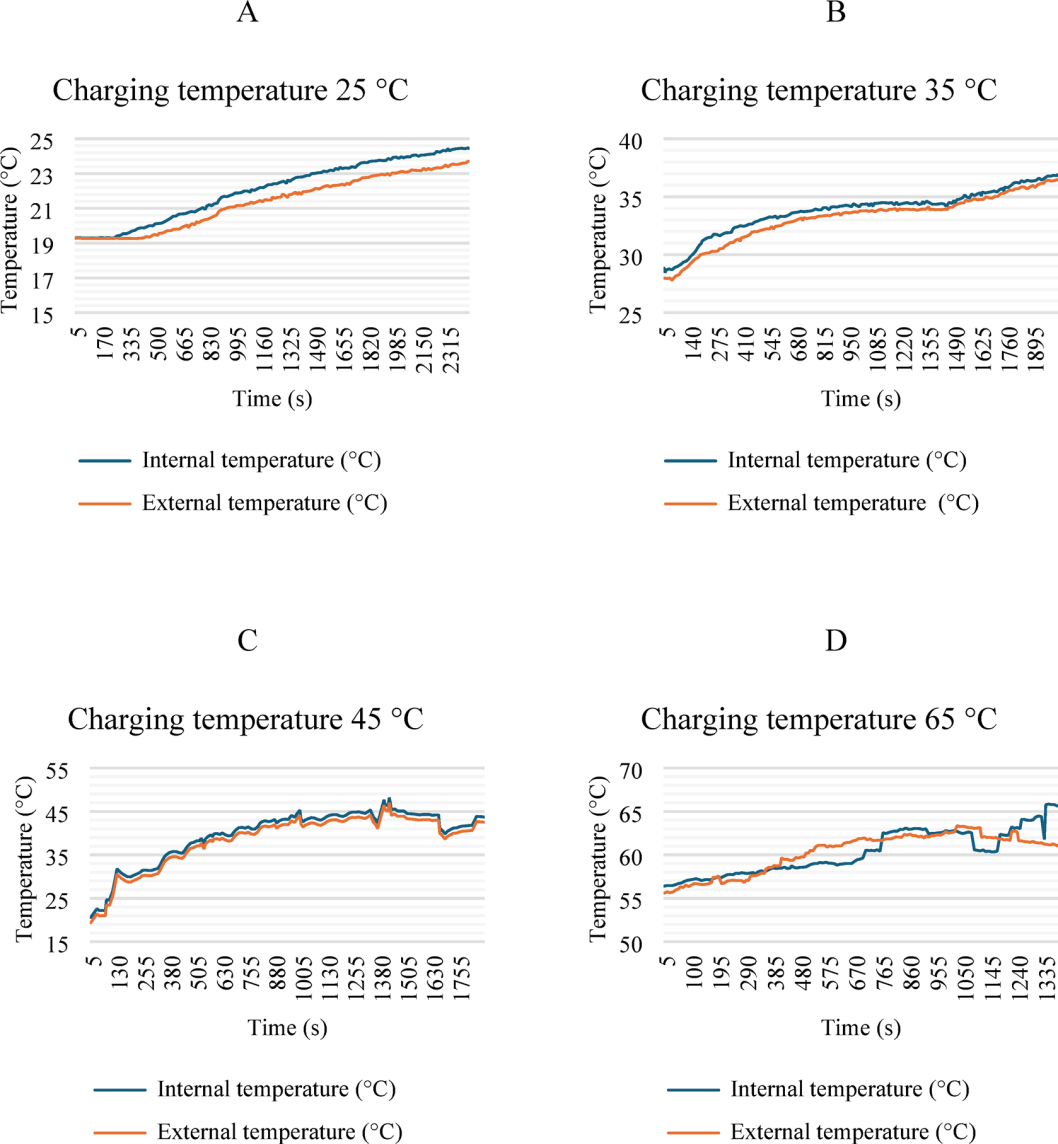
Temperature behavior during the charging process.

Figure 15 shows the temperature behavior of the module during the charging process. The LIB module retains more heat internally while transferring it more easily to the sides. When charging at 65 °C, heating occurs in the BMS components and battery cables, which could cause irreversible damage over a long charging period.

**Fig. 16:**
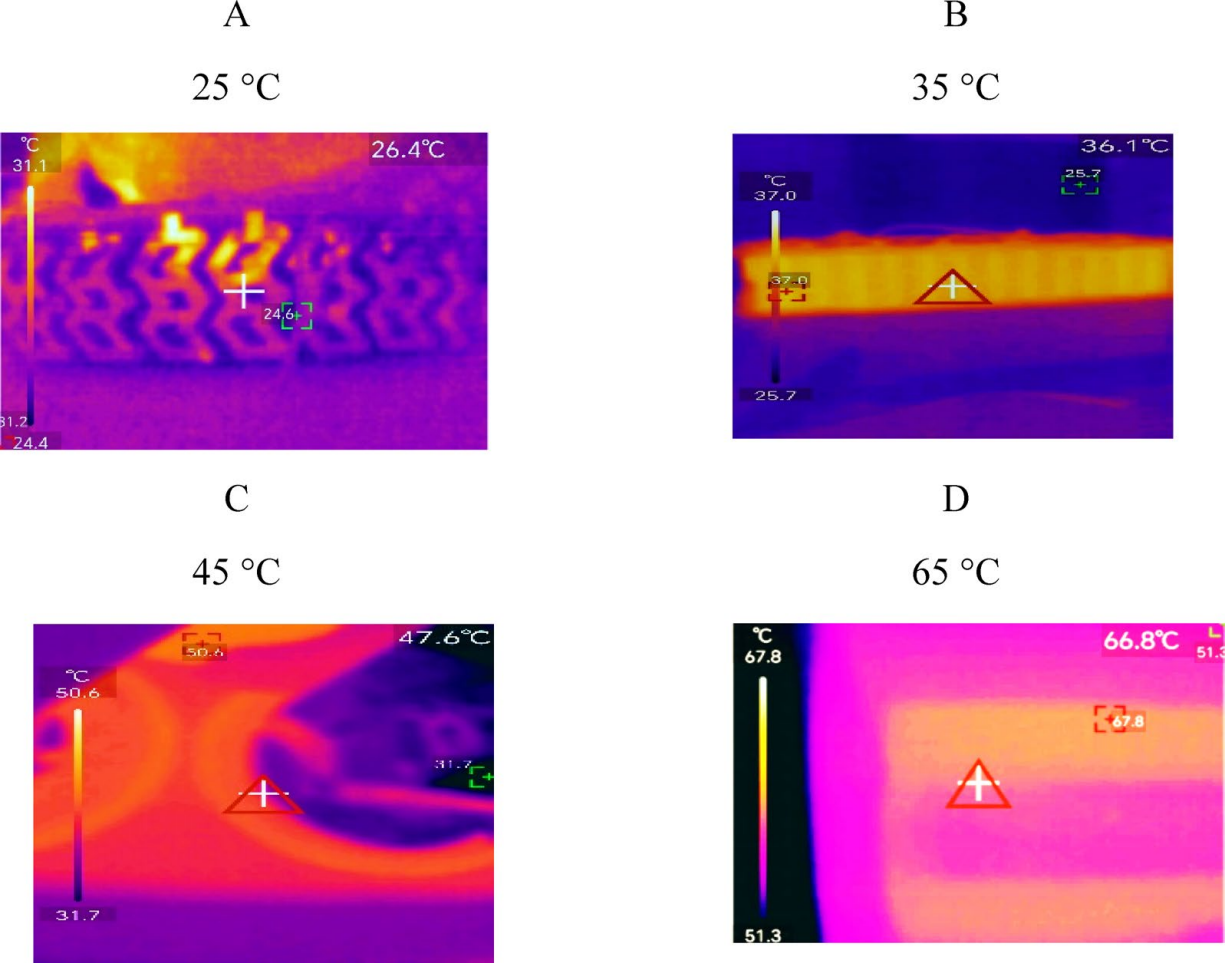
Thermal photographs of the experimental tests.

Figure 16 shows the thermal photographs. It is evident that the highest temperatures are generated in the central part of the module and that the battery components suffer from thermal overheating, which can cause severe damage. In this study, the battery lacks a cooling system, which allows the temperature to be maintained and affects the battery at extreme temperatures. It is also observed that at temperatures of 25 °C and 35 °C, the working temperatures of the battery are within the appropriate range, but above these temperatures the batteries experience a greater increase in temperature, which accelerates their aging.

## Discussion

This study demonstrated that the optimal temperature range for good battery performance is 25 to 35 °C. However, other studies show that a thermal management system is also important. For instance, Wiriyasart et al.^38^ showed a simulation in which fluids absorb heat between cells to lower battery temperature and improve performance. Thermal photographs (Fig. 16) demonstrated that without a cooling system, the module retains heat and is unable to dissipate it, which affects the cell temperature. Nevertheless, Chang et al.^39^ indicated that it is relevant to consider the changes in temperature in the negative and positive poles of the LIB.

There are visible effects that the LIB can suffer, such as deformation, in this study the cells apparently show a healthy state because it requires more time and temperature for them to suffer any deformation as presented in the article by Zhao, et al., where their study is based on temperatures of 80, 90 and 100° C, during a period of 10 h, the cells show that there was discoloration and swelling, in addition to an excess of discolored electrolyte at the edge of the bag^34^. Another negative effect that was shown is the study by Ouyang et al., they subjected the LIB to temperatures of 26 and 70 °C, the LIB showed something of a degradation of load capacity^40^. Mariyam, et al., also demonstrated in their study these findings confirm the degradation of the cells due to high temperatures^41^.

Currently, many countries experience ambient temperatures above 45 °C^42^, which directly affects batteries and, in extreme cases, causes explosion damage. Most LIBs for LEV consisting of a single battery module, lack a cooling system despite being something of great relevance, perhaps this is because technically the size of the LIB is smaller compared to a larger electric vehicle, as mentioned by Cicconi et al.^43^, “the larger the LIB size, the greater the heat production”. However, that study showed that also in the LIB, being a module made up of 52 18,650 type cells, heat is generated and conserved, affecting its performance and useful life.

Likewise, it is important to consider the design of the battery module or pack, this will allow better thermal management as shown in the results by Chung et al.^44^, where different battery arrangements are simulated, showing in most cases that the heat is conserved in the central part of these arrangements, the same conclusion as was obtained when performing the experiment with the LIB module in this study, where thermographic photographs reveal that the heat is concentrated in the cells in the middle part. The concentration of heat in the central part can cause severe damage to the battery module; high temperatures evaporate the electrolyte, causing internal problems in the cell and thus affecting neighboring cells, which can generate a fire. It is considered that cooling systems require more space because they have a fluid and a single module, then an air cooling should be included. In this sense, Adhikari et al.^33^ proposed different cooling systems, concluding that, for small batteries, the air-based systems with external power supply may be more advantageous than conventional systems. SOC and SOH are fundamental parameters in a battery management system (BMS) to optimize the performance, longevity, and safety of the battery.

It was confirmed that heat is generated in the central part of the battery module, as mentioned in the study by The College of Mechanical and Vehicle Engineering Chongqing University, which demonstrated that heat generation directly affects voltage and high temperatures affect battery SOC and SOH^19^. Experimental tests have shown that it is important to have a cooling system that aids heat dissipation, as demonstrated by As Yousefi et al.^11^, who propose fins. Currently, not all LEVs have a cooling system, which can cause irreversible damage to the batteries. Some authors recommend cooling systems, as mentioned by Vakilzadeh et al.^20^, who also confirm that the central part contains a higher concentration of heat. Madani et al.^22^ showed that LIB temperatures outside the recommended range reduce the SOH value, which would lead to improper operation. Since it was shown that the central cells are the most affected, easy access to the cells should be considered; this will help in the replacement of specific cells, thus avoiding a high cost when replacing the entire battery module.

There are aspects of LIBs that are being improved, such as sensitivity to low and high temperatures. Analyzing battery heat transfer is shown to play a fundamental role in achieving better safety and proper battery operation.

It has also been shown that the heat released by one cell directly affects nearby cells through three methods: radiation, conduction, and convection, as demonstrated. The thermal stability is one of the main problems that directly affect safety; at higher temperatures, exothermic reactions occur in the cells, generating heat and accelerating internal cell reactions if there is inadequate heat transfer. This research on the heat generated by lithium-ion batteries (LIBs) focuses primarily on the irreversible heat produced by their internal resistance and the reversible heat produced by electrochemical reaction. Generally, the reversible heat of the reaction is small and can be ignored at room temperature. Therefore, the battery’s heat comes mainly from the irreversible heat produced by the internal resistance. The battery’s internal resistance is affected by multiple factors (state of charge, temperature, discharge rate, etc.).

However, the state of charge (SOC) has a greater influence on the internal resistance at low temperatures because the SOC affects the battery’s resistance value by influencing the rate of LIB´s disintegration and fouling at the anode and cathode, as well as the viscosity of the electrolyte.

## Conclusion

This study emphasized the analysis of LIB´s across a wide temperature range. The proposed method demonstrates reliability under different operating conditions and temperatures of the lithium-ion batteries (LIBs). This was verified using a standardized driving cycle regime and precise thermal imaging. This information supports the development of a mathematical model that can predict the state of charge (SOC) and state of health (SOH) of each battery. The study also examined the voltage behavior of LIBs. Under normal conditions (25 °C and 35 °C), the discharge voltage exhibited stable operating behavior, reaching a maximum voltage of 54.2 V during charging. However, at temperatures of 45 and 65 °C, the voltage level decreased during discharge and the charging processes did not reach the required level for the defined study case application. Some of cells in battery cells exhibited significant capacity loss, reaching levels below 80% SOH. Thermal images showed that the greatest concentration of heat in a battery module is in the central section (F2 and F3, corresponding to cells 5-6-7-8). Most LIBs lack a cooling system that allows for heat dissipation.

The highlights of this study are:

1. The higher the temperature of the LIB’s operating environment, the greater the voltage loss it will experience, resulting in lower autonomy in the LEV (SOH).
2. The central cells (F2 and F3, cells 5-6-7-8) retain a higher temperature, which necessitates the implementation of a ventilation system that operates in accordance with the power energy demands.
3. High temperatures affect the module’s state of charge because extreme temperatures increase the internal resistance of the LIB’s.
4. When the LIB environment is at a high temperature, the BMS causes a cut-off during charging, which in some cases leads to an imbalance in the cell charge and, in other cases, reduces their SOC.

In the experiment, it was observed that some cells did not have a 100% SOH, which could be attributed to various factors, including transportation and prolonged storage periods. It was demonstrated that a LIB module suffers various damages caused by high temperatures, and the reduction in the vehicle’s range is evident. During the charging process, a cell imbalance was observed, caused by the BMS protection system. These experimental tests were conducted in a safe location, avoiding any possible implosion due to thermal shock. It is essential to note the precautions taken, including maintaining clean work surfaces, avoiding short circuits, and considering the capacity of materials (such as resistors, cables, and electronic circuits) to prevent overloads and damage to the elements. The importance of analyzing the LIB cell by cell was highlighted; this allowed for the precise identification of lithium-ion batteries (LIB) with the greatest wear, considering the state of charge (SOC) and state of health (SOH) values. As possible future work, it is necessary to emphasize a battery thermal strategy (BTS), with a comprehensive approach that manages the optimal temperature cell by cell.

## Supplementary Information

Below is the link to the electronic supplementary material.

Supplementary Material 1

## Data Availability

The data used to support the findings of this study are available from the corresponding author upon request.
