# Supplementary material for: Temperature and voltage effects on the charge and health of lithium-ion battery modules in light electric vehicles
Source: Sci Rep. 2026 Feb 17;16:9408. doi: 10.1038/s41598-026-40094-5 (PMC13003025; doi:10.1038/s41598-026-40094-5)
Supplement: Supplementary file 1 — Supplementary Material 1 [file 41598_2026_40094_MOESM1_ESM.docx]

**Nomenclature**

| **Nomenclature** | |
| --- | --- |
| W | Watt |
| V | Volt |
| Ah | Ampere hour |
| °C | Celsius degress |
| h | Hour |
| *Q_cu_* | Current maximum available capacity |
| *Q_re_* | Remaining capacity |
| *Q_e_* | Rated capacity |
| Km/h | Kilometres per hour |
| Kg | kilograms |
| s | Seconds |
| cm | Centimeters |
